# Recent advances of cell membrane-coated nanoparticles for therapy of bacterial infection

**DOI:** 10.3389/fmicb.2023.1083007

**Published:** 2023-02-17

**Authors:** Yue Song, Xia Zheng, Juan Hu, Subo Ma, Kun Li, Junyao Chen, Xiaoling Xu, Xiaoyang Lu, Xiaojuan Wang

**Affiliations:** ^1^Stomatology Hospital, School of Stomatology, Zhejiang University School of Medicine, Key Laboratory of Oral Biomedical Research of Zhejiang Province, Hangzhou, China; ^2^The First Affiliated Hospital, School of Medicine, Zhejiang University, Hangzhou, China; ^3^Shulan International Medical College, Zhejiang Shuren University, Hangzhou, China

**Keywords:** cell membrane, biomimetic nanoparticles, antibiotics delivery, bacterial infection, bacterial resistance

## Abstract

The rapid evolution of antibiotic resistance and the complicated bacterial infection microenvironments are serious obstacles to traditional antibiotic therapy. Developing novel antibacterial agents or strategy to prevent the occurrence of antibiotic resistance and enhance antibacterial efficiency is of the utmost importance. Cell membrane-coated nanoparticles (CM-NPs) combine the characteristics of the naturally occurring membranes with those of the synthetic core materials. CM-NPs have shown considerable promise in neutralizing toxins, evading clearance by the immune system, targeting specific bacteria, delivering antibiotics, achieving responsive antibiotic released to the microenvironments, and eradicating biofilms. Additionally, CM-NPs can be utilized in conjunction with photodynamic, sonodynamic, and photothermal therapies. In this review, the process for preparing CM-NPs is briefly described. We focus on the functions and the recent advances in applications of several types of CM-NPs in bacterial infection, including CM-NPs derived from red blood cells, white blood cells, platelet, bacteria. CM-NPs derived from other cells, such as dendritic cells, genetically engineered cells, gastric epithelial cells and plant-derived extracellular vesicles are introduced as well. Finally, we place a novel perspective on CM-NPs’ applications in bacterial infection, and list the challenges encountered in this field from the preparation and application standpoint. We believe that advances in this technology will reduce threats posed by bacteria resistance and save lives from infectious diseases in the future.

## 1. Introduction

Since its discovery in 1970s, antibiotics have been utilized to combat microbes and protect people from fatal infections. However, the overuse of antibacterial agents and the mutations in bacteria have caused significant drug resistance(Kurt Yilmaz and Schiffer, 2021; Saha and Sarkar, 2021). However, the process of developing novel antibiotics is extremely expensive and time-consuming. The development of novel potent antibiotics is still lagging (Wang X. et al., 2022). Novel antibacterial agents emerge much slower than bacteria resistant to them. Bacterial infections are expected to cause 10 million deaths per year by 2050. Approximately $100 trillion can be spent on treating drug-resistant infections globally (Zhou et al., 2021). Consequently, superbugs have emerged as a major health challenge (Church and McKillip, 2021). In a variety of scientific fields and in pharmaceutical companies, researchers have explored for novel antibiotics and therapeutic strategies to overcome bacterial resistance.

Aside from drug resistance, toxins pose a major challenge in the clinical management of bacterial infections (Escajadillo and Nizet, 2018; Keller et al., 2020). During the process of antibiotics killing bacteria, their exotoxins and endotoxins are rarely eliminated. The rupture of the bacteria may produce more endotoxins, further causing harm to the body (Ma et al., 2022). Moreover, a close relationship exists between the pharmacokinetics (PK) and pharmacodynamics (PD) properties of antibiotics. For example, meropenem exhibits time-dependent PD property. The time that free drug concentrations remain above the minimum inhibitory concentration (MIC) as a function of the dosing interval (%fT > MIC) has been shown to best predict antibacterial effect, in which MIC refers to the lowest concentration of drug capable of inhibiting bacterial growth (Pascale et al., 2019; Liu J. et al., 2021). Clinically, shortening the dosing interval, prolonging the infusion time, and increasing the dosage has been utilized to increase the %fT > MIC. However, a sole focus on optimizing drug dosing regimens offers limited results. Furthermore, pathogenic bacteria can form biofilms on the surfaces of medical devices or in infection sites. A biofilm’s dense and adhesive structure can severely reduce the effectiveness of drug enrichment by preventing drug penetration (Gebreyohannes et al., 2019). Biofilms are also excellent for horizontally spreading antibiotic resistance genes (Bowler et al., 2020). Antibiotic resistance is aggravated by these issues, and high doses of antibiotics potentially causes organ damage. In summary, conventional antimicrobials are inadequate for the successful treatment of bacterial infections, particularly those caused by multidrug-resistant bacteria.

Nanodrug delivery systems (DDSs) can increase the retention time in the circulation, reduce the non-specific distribution, and enable targeted delivery of drug to the infection sites (Wang X. et al., 2022). Thus, the use of DDSs for treating deadly infections has recently been found to be a promising therapeutic possibility (Gupta et al., 2019; Makabenta et al., 2021). Combinations of nanomaterials and antibiotics may enhance therapeutic efficacy. As a result, they provide promising therapeutic options for combating bacteria and treating infectious diseases. Au, Ag, Cu, Fe and Ti-based DDSs have been developed for treating infectious diseases, but their clinical use is held back by safety concerns. The metallics may leak out and harm people as well (Guo et al., 2020). The development of smart nanomaterial-based delivery strategies, such as pH-activated, enzyme-activated, and bacterial toxin-activated DDSs, have attracted considerable interest (Canaparo et al., 2019). However, their sophisticated fabrication process has hindered their designs from entering the large-scale production stage. The body also has difficulty in metabolizing the complex material. Therefore, novel systems are urgently needed to overcome the above issues.

The use of nanoparticles (NPs) modified by natural cell membranes or synthetically produced membranes, known as “biomimetic nanoparticles,” has advanced the fields of drug delivery and received considerable attention in recent years (Fang et al., 2018). Cell membrane-coated nanoparticles (CM-NPs) combine some of the best features of both host and artificial nanoparticles (Zhen et al., 2019). Owing to their unique features, such as immunological invasion and enhanced targeting capacity, CM-NPs confer significant therapeutic and diagnostic value (Imran et al., 2022b). Another benefit of CM-NPs is their ability to preserve the intrinsic features and abilities of cell membranes. The biocompatibility of CM-NPs is good because they are considered by the body as parts of itself. Thus, since Zhang et al.’s report on cell membrane-coating technology in 2011, numerous membrane-mimicking nanoplatforms have been constructed for biomedical applications, with a primary emphasis on cancer therapy (Imran et al., 2022a).

It has also been reported that CM-NPs are capable of evading immune recognition, targeting pathogenic bacteria, neutralizing toxins, and delivering antibiotics for combating bacterial infections (Rao et al., 2020; Wang et al., 2020). Currently, the membranes of erythrocytes, platelets, macrophages, neutrophils, epithelial cells, bacterial and hybrid membranes have all been successfully applied for CM-NPs preparation (Ma et al., 2022). Due to their various constituents, such as membrane proteins, glycans, and lipids, cell membranes from various origins serve distinct functions. For example, nanoparticles coated with erythrocyte membranes have a longer circulation half-life time than those modified by polyethylene glycol (PEG) because they inherit the ability of red blood cells (RBCs) to circulate blood for an extended duration (Fan et al., 2020). White blood cells (WBCs) membrane-coated biomimetic nanoparticles are endowed with immune evasion and inflammatory chemotaxis functions (Wang D. et al., 2022). Moreover, Yingying Gan et al. propose the strategy of “fight bacteria with bacteria.” The potential of membrane vesicles (MVs) derived from bacteria as delivery systems to treat bacterial infection has been extensively researched as well (Gan et al., 2021). This review will provide an overview of the methods used in preparing CM-NPs, examining the obstacles and future directions faced by the field, and providing an outlook on the potential benefits to patients.

## 2. Preparation of cell membrane-coated nanoparticles

The preparation of CM-NPs mainly consists of three steps: membrane extraction, core nanoparticle synthesis, and fusion. All these steps are important for maintaining the intended functions of biomimetic nanoparticles (Liu et al., 2019).

Phospholipids and certain surface proteins constitute cell membranes. Membranes often have pivotal role in different types of biological processes. Extracting cell membranes requires two critical steps: membrane lysis and membrane purification (Fang et al., 2018). Both of the process require pinpoint accuracy and careful handling. Various membranes determine the specific extraction procedure. Cells without nuclei, such as mature RBCs and platelets, make membrane extraction a straightforward operation. After cells are separated from whole blood, the membranes are forcefully disrupted through hypotonic lysis or repeated freezing and thawing. Then, soluble proteins are separated through differential centrifugation, and nanovesicles are extruded into their final shapes. Obtaining membrane materials from eukaryotic cells, such leukocytes, requires elaborate methods. Isolating desired cells from the blood or tissues and culturing the cells are the initial steps. Then, cell membranes are isolated by removing nuclei and cytoplasm *via* hypotonic lysis, mechanically disrupting the membrane, and centrifugation of the pellet in a discontinuous sucrose gradient. Isoionic buffers clean the membranes before sonication and extrusion through the membranes’ porous polycarbonate matrices. In addition, MVs and exosomes are usually extracted by removing dead cells and cellular debris *via* centrifugation. Subsequently, evenly sized exosomes and bacterial MVs are obtained through ultrahigh-speed centrifugation (Liu et al., 2019; Ma et al., 2022).

Inner nanoparticles are crucial to biomimetic nanomaterial production because they carry antimicrobial components to sick tissues. Recently, the use of different materials encapsulated by cell membranes, such as mesoporous silica nanocapsules, metal–organic frameworks (MOFs), gold nanoparticles, nanogels, nanocrystals, and poly(lactic-co-glycolic acid) (PLGA) nanoparticles, have been explored and developed (Fang et al., 2018). The preparation processes vary by nanomaterial and delivery cargo requirement.

After cell membranes and inner core nanoparticles are obtained, CM-NPs undergo a membrane coating process on the surfaces of the nanoparticles. Current fusing techniques include membrane extrusion, ultrasonic fusion, electroporation, and co-incubation (Fang et al., 2018; Imran et al., 2022a). Membrane extrusion and ultrasonic fusion are the two most often employed techniques. In membrane extrusion, mechanical forces enable nanoparticles and cell membranes to traverse membranes with varying pore diameters, allowing the coating of nanoparticles by cell membranes. However, despite being practical and effective, this technique is difficult to use in large-scale production. Similar to physical extrusion, ultrasound results in the spontaneous formation of a core-shell structure between membranes and nanoparticles with diminished material loss. However, resulting particles may exhibit large size heterogeneity and lack of homogeneity. The nanoparticles can be damaged by ultrasonography. A unique microfluidic electroporation strategy has recently been used for the production of membrane-coated nanoparticles. In this method, ingredients are combined in a Y-shaped or S-shaped conduit, where they are thoroughly mixed before electroporation. The method has been utilized to generate nanoparticles with high yields and high reliability after suitable tuning. Another approach for obtaining NP-containing exosomes is to incubate live cells with nanoparticles, and then induce secretion in a serum-free media. These procedures differ from the coating procedures described above, which entail purifying the cell membrane [!!! INVALID CITATION!!! (Ma et al., 2022)]. An ideal coating process should produce stable nanoparticles with consistent size and shape without altering the membranes’ or core particles’ functional qualities.

## 3. Cell membrane-coated nanoparticles for bacterial infection treatment

### 3.1. Red blood cell membrane-coated nanoparticles

As a natural bio-membrane isolated from RBCs, the RBC membrane (RBCM) has attracted substantial interest as a potential drug carrier (Jin et al., 2018; Xia et al., 2019). Immunomodulatory indicators in the RBCM, such as CD47, sialic acid, peptides, and glycans, prolong the circulation of medicines by reducing absorption by macrophages (Gao et al., 2013; Chen et al., 2020). Liangfang Zhang et al. revealed that RBCM modification decreased the cellular uptake of PLGA nanoparticles by macrophages, and PLGA nanoparticles exhibited a longer half-life time than the widely applied long-circulating PEG-modified nanoparticles (Hu et al., 2013). Bacteria can create life-threatening toxins. Endotoxins and exotoxins are the major sources of bacterium-induced cytokine outbursts. Traditional detoxification methods rely on antigen–antibody specific binding, and each antibody is designed to neutralize a specific antigen (Ma et al., 2022). A challenge encountered in these methods is determining the structures of toxins and pathogenic microorganisms. Methods for neutralizing toxins disarm pathogens and directly ameliorate infection symptoms due to their destructive nature and crucial functions in pathogen processes (Zou et al., 2021). RBCs can be destroyed by various bacterial exotoxins, and RBC membrane-coated nanoparticles, sometimes known as“nanosponges,“can serve as decoy targets for several different bacterial toxins (Wang S. et al., 2022). Owing to the high affinity between the RBCM and exotoxins, nanosponges show great potential in neutralizing a wide variety of toxins. Additionally, the RBCM is considerably simple to isolate and purify because mature RBCs have no nuclei or other organelles. Thus, RBCM-modified nanoparticles fighting bacterial infections have drawn the attention of researchers. Representative RBCM-coated nanoparticles for bacterial infection treatment are listed in Table 1.

**Table 1:** Representative red blood cell (RBC) membrane coated nanoparticles for therapy of bacterial infection.

Core materials	Functions	Pathogens	Infection model	References
PLGA nanoparticle	Sequester SLO; block the ability of GAS to damage host cells	GAS	Necrotizing skin infection	Escajadillo et al. (2017)
PLGA nanoparticle	Attenuates β-H/C-mediated hemolysis, cell death and apoptosis; suppress neutrophil bactericidal properties, and inflammasome activity	GBS	None	Koo et al. (2019)
Chitosan-functionalized PLGA nanoparticles	Neutralize toxin; prolong retention time	GAS	Subcutaneous infection	Zhang et al. (2017)
Fe_3_O_4_ nanoparticles	Absorb toxins; kill bacteria with photothermal effect	MRSA	Wound infection	Chen et al. (2021)
PLGA nanoparticle encapsulating tedizolid	Escaping immune system; neutralize exotoxins; excellent biocompatibility	MRSA	Wound infection	Wu X. et al. (2021)
Perfluorocarbons loading therapeutic gases like oxygen or NO	Neutralize toxins; pore-forming toxin responsive release of photosensitizer, oxygen and NO; realize “on-demand” photodynamic therapy and kill bacteria	MRSA, GAS, *Listeria monocytogenes*	Skin infection	Zhuge et al. (2022)
Supramolecular gelatin nanoparticles loading vancomycin	Evade immune system; absorbs toxins; site-specifically release of antibiotics and kill bacteria	MRSA	None	Li et al. (2014)
Gelatin nanoparticles encapsulating Ru − Se nanoparticles	Evade immune system; absorbs toxins; the responsive release of Ru-Se nanoparticles; provide real-time *in vivo* imaging	MRSA, *E. coli*	MRSA-infected mice knife injury model	Lin et al. (2019)

PLGA, poly(lactic-co-glycolic acid); GAS, group A *Streptococcus*; SLO, GAS pore-forming streptolysin O; GBS, Group B *Streptococcus*; β-H/C, β-hemolysin/cytolysin; MRSA, methicillin-resistant *Staphylococcus aureus*; NO, nitric oxide; *E. coli*, *Escherichia coli*.

**Figure 1:**
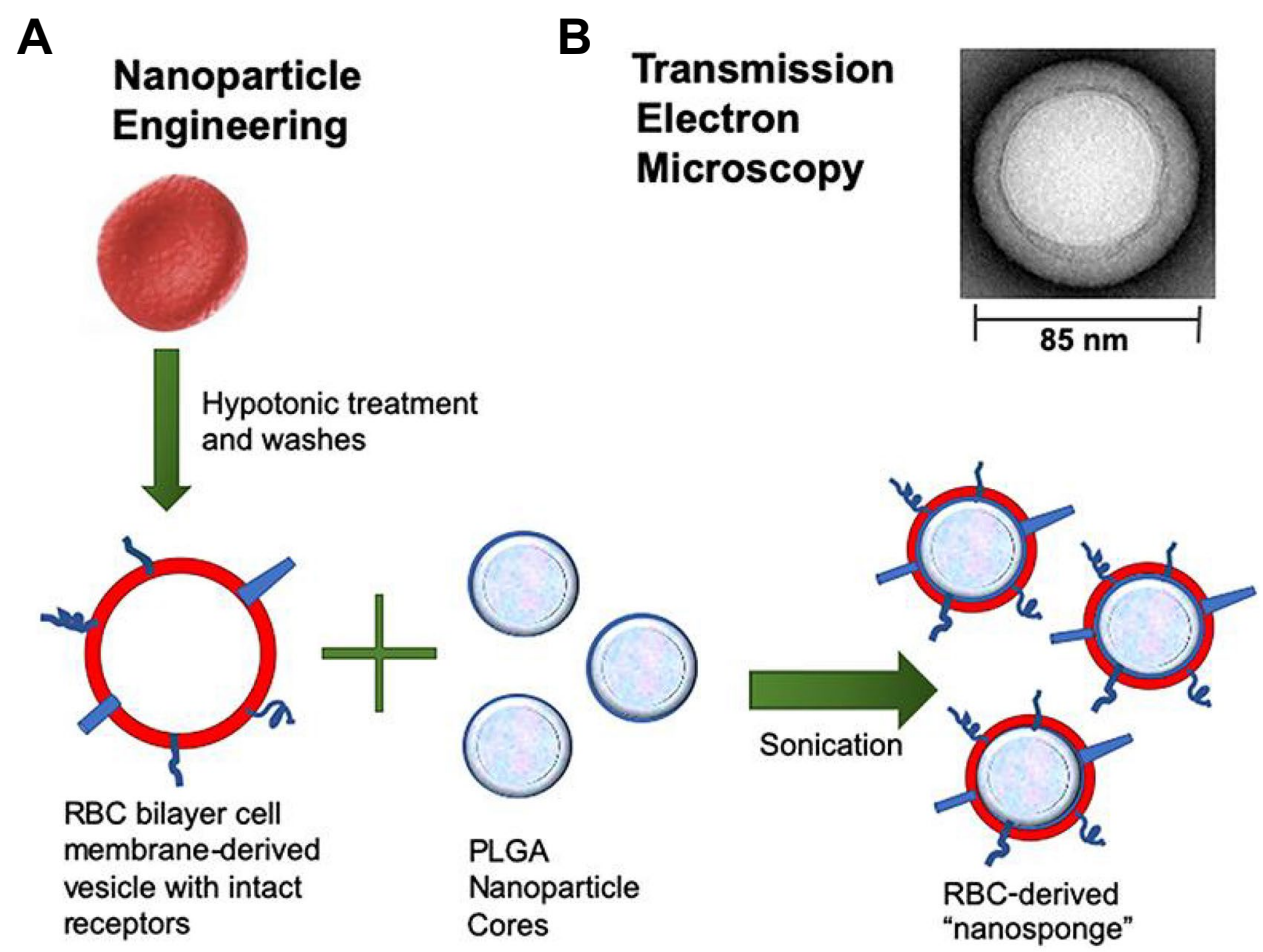
Preparation process of RBCM-derived nanosponges. **(A)** Schematic diagram of the fusion process of erythrocyte membrane and PLGA nanoparticles into nanosponge. **(B)** Transmission electron microscopy image of a single toxin-absorbing nanosponge. (Koo et al., 2019). RBCM, red blood cell membrane; PLGA, poly(lactic-co-glycolic acid).

Biomimetic nanoparticles composed of ~100 nm PLGA nanoparticle core and RBCM were developed for infection induced by Group A *Streptococcus* (GAS). The constructed nanosponges sequestered streptolysin O (SLO), a well-defined virulence factor generated by the vast majority of GAS, and prevented GAS from damaging host cells, thus maintaining innate immune function and enhancing bacterial clearance. Neutrophils, macrophages, and keratinocytes were protected from SLO-mediated cytotoxicity after treatment with nanosponges. The topical use of biomimetic nanosponges reduced the lesion size and the counts of bacterial colony-forming units in mice with GAS necrotizing skin infection (Escajadillo et al., 2017). The constructed nanosponges (the preparation process shown in Figure 1) significantly blocked the cytotoxic effects of pore-forming toxin β-hemolysin/cytolysin (β-H/C) of Group B *Streptococcus* (GBS). Nanosponge therapy prevented cellular death in lung epithelial cells and macrophages after exposure to GBS. In addition, increased GBS killing by neutrophil and lowered levels of IL-1β produced by macrophages were observed in response to GBS. In general, nanosponges exhibited a negative charge due to the nature of cell membranes, and could interact with cationic compounds (Koo et al., 2019). Zhang et al. mixed nanosponges with chitosan-functionalized PLGA nanoparticles possessing a similar size but opposite surface charge. After mixing, the two types of nanoparticles formed a stable three-dimensional network, dubbed a “nanosponge colloidal gel” (NC-gel). The gel preserved RBC-nanoparticles in the network without affecting their toxin-neutralizing capacity. The obtained NC-gel, an injectable preparation, demonstrated remarkable antibacterial capacity as reflected by the significantly reduced skin lesions in mice subcutaneously infected by GAS (Zhang et al., 2017).

Infections caused by bacteria typically result from released or secreted toxins. The process of toxin absorption by RBCM-coated nanoparticles seems like disarming the “enemy,” thus relieving clinical symptoms. However, the bacteria, the enemy without power, are not killed or destroyed. The strategy of “disarming” and “killing” the enemy simultaneously may be a means for treating infections effectively. Methicillin-resistant *Staphylococcus aureus* (MRSA) has garnered considerable interest as a major drug-resistant pathogen for causing soft tissue infections, pneumonia, bacteremia, and other deadly illnesses. Unfortunately, the therapeutic efficacy of vancomycin (Van), the well-known final line of defense against MRSA infections, is diminishing year after year (Rani et al., 2021). Chen et al. reported that biomimetic recycled RBC@Fe_3_O_4_ nanoparticles are antibacterial agents. In addition to toxin-absorbing activity, the prepared nanoparticles demonstrated bacterium-damaging capability through a photothermal effect under near infrared light irradiation. The collaborative treatment resulted in excellent therapeutic effect in mice with MRSA-infected wound (Chen et al., 2021). In another study, RBCM-coated PLGA nanoparticles were used for tedizolid phosphate delivery. The prepared nanoformulation displayed excellent compatibility and accelerated the healing rate of mice with MRSA-infected wound (Wu X. et al., 2021).

**Figure 2:**
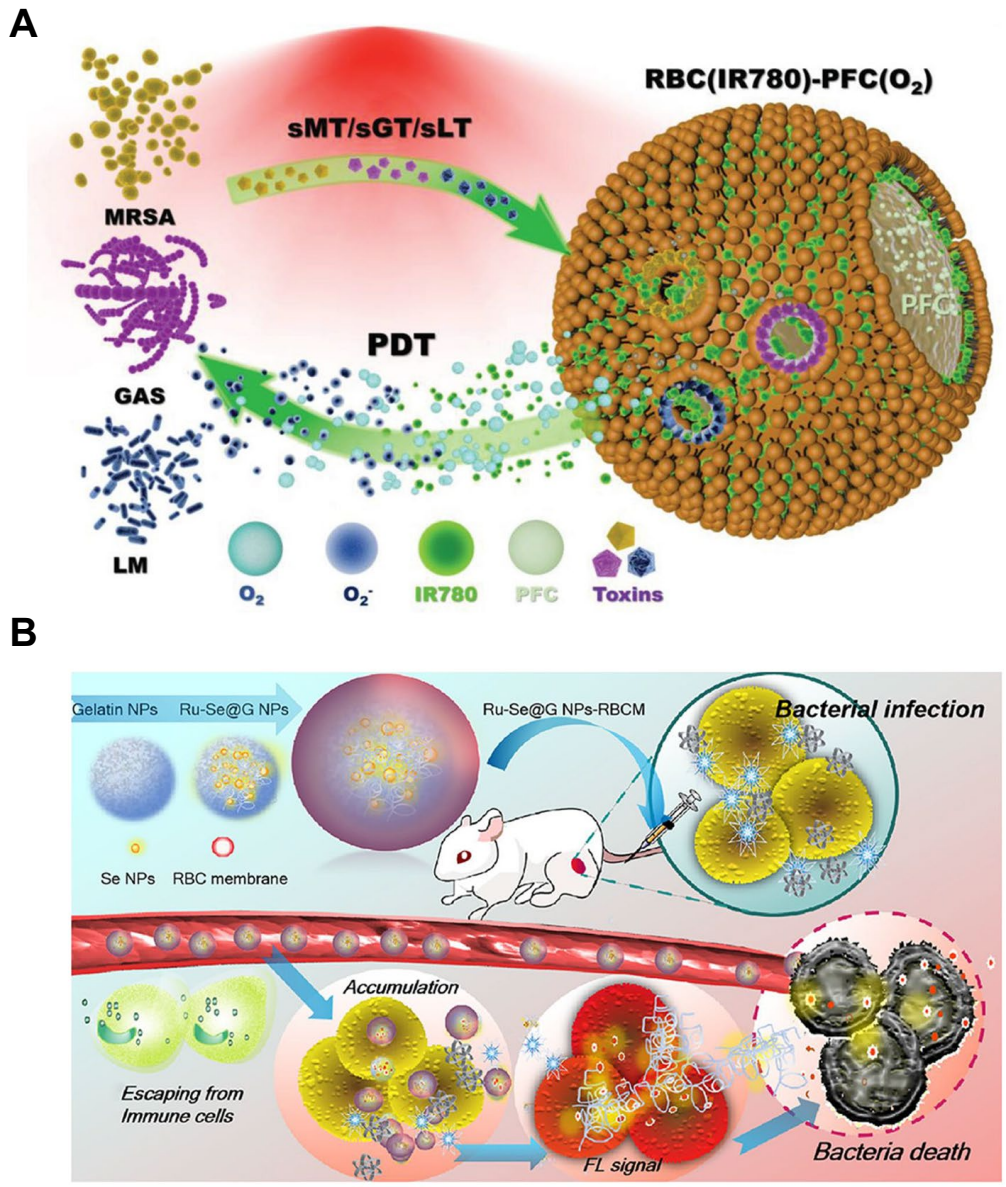
Schematic diagrams of microenvironment-responsive RBC membrane-coated nanosystems. **(A)** “On-demand” photodynamic therapy (PDT) of bacterial infection. The constructed RBC(IR780)-PFC(O_2_) nanobubbles can bind to and neutralize toxins secreted by MRSA, GAS, and LM (named sMT, sGT, and sLT, respectively). After absorbing toxins, the formed pore stimulated the release of the photosensitizer IR780 and oxygen. Subsequently, the released agent contributed to the enhancement of PDT efficiency in killing bacteria under NIR irradiation. Reprinted with permission from Zhuge et al. (2022) (Copyright© 2022, Wiley-VCH). **(B)** The prepared Ru − Se@GNP-RBCMs can escape from immune cells, accumulate at infectious sites, and realize responsive release of Ru − Se nanoparticles. The synergistic antibacterial effect finally resulted in the bacterial death. Reproduced with permission (Lin et al., 2019; Copyright© 2019, American Chemical Society). MRSA, methicillin-resistant *Staphylococcus aureus*; GAS, Group A *Streptococcus*; LM, *Listeria monocytogenes*; NIR, near infrared; GNP, gelatin nanoparticles.

As a system for biomimicry, RBCM-coated nanoparticles effectively counter pore-forming toxins (PFTs) produced by different types of bacteria. Without inducing the development of antibiotic resistance, photodynamic therapy (PDT) shows great promise in treating multidrug-resistant microorganisms. Based on these developments, an RBCM-coated PFT-responsive nanobubble system was developed for the on-demand release of therapeutic gases and therapy of bacterial infections. RBCM coating allowed nanobubbles to bind and neutralize several types of PFTs, thus preventing toxin-mediated cytotoxicity from affecting healthy cells. By forming pores on nanobubble surfaces, therapeutic cargoes can be rapidly released within the microenvironments of bacterial infections, thereby significantly improved antibacterial capacity. In O_2_ delivery, supplied oxygen increases PDT *via* the generation of reactive oxygen species after laser irradiation. Moreover, additional therapeutic gases, such as nitric oxide, can be delivered and released in a bacterial toxin–dependent manner (Figure 2A; Zhuge et al., 2022). This research provided a general strategy for the on-demand delivery and release of therapeutic gases and for improving precision in the treatment of bacterial infections.

Extremely diverse microorganisms produce gelatinase (Yang et al., 2022). Biocompatible and degradable gelatin nanoparticles (GNPs) can serve as optimal drug delivery vehicles for the microenvironment-responsive release of encapsulating antimicrobial agents. In addition to PFT-responsive nanosytems, RBCM is applied to bacterium-responsive biomimetic nanosystems. For example, core–shell supramolecular GNP coated with RBCM was constructed for adaptive and “on-demand” Van delivery. Given that the core consisted of cross-linked GNPs, the encapsulating Van was released in a microenvironment-responsive manner. The shell RBCM coating served as a mask that inhibited clearance by the immune system during antibiotic delivery (Li et al., 2014). The RBCM absorbed bacterial exotoxins, thus alleviating the symptoms of bacterial infection (Zhuge et al., 2022). This design illustrated a novel antibiotic delivery system for treating bacterial infection at a low drug dose. However, the therapeutic effect of the constructed nanosystems *in vivo* has not been investigated. In another study, Ange Lin et al. reported an RBCM-coated GNP for delivering Ru complex–modified selenium nanoparticles (Ru-Se NPs). The developed nanoformulation Ru-Se@GNP-RBCM showed the following advantages: (1) the RBCM exerted immune-evading and toxin-clearance capacities; (2) the degradation of GNP by gelatinase facilitated the responsive release of Ru-Se NPs in infectious microenvironments; (3) the nanosystem exerted synergistic antibacterial activity and real-time *in vivo* imaging capacity, allowing the accurate monitoring of the treatment procedure (shown in Figure 2B; Lin et al., 2019). The presented results confirmed the hypothesis. However, the process of preparing the nanosystem is complicated, and additional characterization is needed for confirming successful synthesis. Additionally, its effectiveness against bacteria is not greater than that of Van. Nevertheless, the reported biomimetic nanoparticle Ru-Se@GNP-RBCM still exhibited remarkable potential as a biological antibiotic replacement.

The generation of biofilms impedes the entry of antibiotics and deactivates them, consequently developing bacterial resistance (Malaekeh-Nikouei et al., 2020). RBCM-NPs displays potential in combating biofilm as well. For example, a novel biomimetic nanosystem, RBCM-NW-G, composed of RBCM, nanoworm (NW) particles and gentamicin was developed by Ran et al. The obtaining nanoparticles retained the capacity of RBCM with a longer blood circulation time and good biocompatibility. AuAg and polydopamine in the nanoworm exerted photothermal effect under near infrared excitation. Near infrared excitation triggered the release of antibiotic and Ag^+^ into the microenvironment. The increased loacal temperature damaged the biofilms. Subsequently, antibiotic and Ag^+^ could effectively penetrate the biofilm and achieve excellent bactericidal activity. The research provides a novel means for combating bacterial biofilm infections (Ran et al., 2021).

### 3.2. White blood cell membrane-coating nanoparticles

WBCs, also known as leukocytes, are immune cells that help the body fight off infections, engulf and digest foreign invaders, heal damaged tissues, and ward off illnesses (Wang D. et al., 2022). WBCs, such as macrophages, neutrophils, T cells, and natural killer cells, play crucial roles in bacterial infection and inflammation (Liu et al., 2020). A sentinel monocyte or macrophage recognizes endotoxins released by bacteria during sepsis. Septic shock or death may be cause by these cells activating or potentiating downstream inflammation cascades. Dynamic and varied functions have served as the basis for constructing WBC membrane–coated nanoparticles (WBC-NPs) (Mohale et al., 2022). With wide bio-interfacing properties, WBC-NPs mimic the broad biofunctional properties of source cells and can be used therapeutically. WBC-NPs can recognize the pathogen-associated molecular patterns of harmful bacteria; this ability enables them to realize targeted antibiotic delivery effectively. The benefits of WBC-NPs have led to their investigation as a medication delivery carrier for bacterial infection treatment. Table 2 lists some examples of the WBC-NPs’ uses in treating bacterial infections.

**Table 2:** Representative white blood cell (WBC) membrane coated nanoparticles for therapy of bacterial infection.

Membrane type	Core materials	Functions	Pathogens	Infection model	References
*S. aureus*-pretreated macrophage membrane	Gold–silver nanocage	Target bacteria; prolong retention time; kill bacteria under NIR laser irradiation; good biocompatibility; serve as antibiotics delivery vehicle	*S. aureus*	Subcutaneous infection; *S. aureus*-induced osteomyelitis	Wang et al. (2018)
Bacterially activated macrophage membrane	Silicon nanowires	Capture broad-spectrum bacteria; extracorporeal cleansing devices	ESKAPE	None	Liu S. et al. (2021)
Macrophage membrane	Zn^2+^-based MOF loading antimicrobial gene LL37	Sequester pro-inflammatory cytokines; homotypic targeting gene delivery; continuous production of LL37; eradicate bacteria hidden in macrophage and circulation	MDRSA	Immunosuppressed mouse model with sepsis	Cao et al. (2022)
Macrophage membrane	PLGA nanoparticle	Bind and neutralize endotoxins; sequester proinflammatory cytokines; inhibit the sepsis cascade	*E. coli*	Bacteremia, sepsis	Thamphiwatana et al. (2017)
Macrophage membrane	Magnetic Fe_3_O_4_, Ca_3_(PO_4_)_2_ and TiO_2_	Recognize bacteria, adsorb bacteriotoxin and inflammatory cytokines; inhibit the inflammation; induce bone tissue regeneration; kill bacteria under ultraviolet irradiation	MRSA	Osteomyelitis	Shi et al. (2021)
Macrophage–Monocyte Membrane	A binary, amphiphilic conjugate consisting of ciprofloxacin and triclosan	Selective entry into infected macrophages kill staphylococci internalized in macrophages	*S. aureus*	Peritoneal infection; organ infection	Li et al. (2020)
Neutrophil membrane	Algae-nanoparticle hybrid microrobot loading ciprofloxacin	Fast moving speed in simulated lung fluid; uniform lung distribution; low clearance by alveolar macrophages and superb tissue retention time; specific binding with pathogens.	*P. aeruginosa*	Acute pneumonia	Zhang et al. (2022)
Neutrophil membrane	GOx/CPO-embedded ZIF-8 nanoparticles	Construct host defense against infection; target inflammation; generate reactive HClO to eradicate infection	*S. aureus*	Subcutaneous injection	Zhang et al. (2019)

*S. aureus*, *Staphylococcus aureus*; NIR, near-infrared; ESKAPE, *Enterococcus faecium*, *Staphylococcus aureus*, *Klebsiella pneumoniae*, *Acinetobacter baumannii*, *Pseudomonas aeruginosa*, and *Enterobacter*; MOF, metal–organic framework; MDRSA, multidrug resistant *S. aureus*; PLGA, poly(lactic-co-glycolic acid); *E. coli*, *Escherichia coli*; MRSA, methicillin-resistant *Staphylococcus aureus*; *P. aeruginosa*, *Pseudomonas aeruginosa*; GOx, glucose oxidase; CPO, chloroperoxidase; HClO, hypochlorous acid.

Macrophages are crucial immune cells identifying infections and responding to them. By producing complicated receptors (especially Toll-like receptors), which bind to microbial molecular patterns, macrophages constitute the first line of defense against bacterial infections (Robinson et al., 2019; Galli and Saleh, 2020). Changes in receptor expression and dimerization occur in response to macrophage activation by various microorganisms (Rosenberg et al., 2022). Additionally, when macrophages are cultured with certain bacteria, the expression of recognition receptors on bacterial membranes increases dramatically (Sica et al., 2015). Therefore, macrophage membranes’ unique capacity to identify microorganisms has served as an inspiration for the construction of macrophage-coated nanoparticles. Wang et al. reported a *S. aureus–*pretreated macrophage membrane-coated gold-silver nanocages (Sa-M-GSNC). Their results demonstrated that *S. aureus* pretreatment increased the adhesion capacity of the system to therapeutically relevant bacteria effectively. Apart from heat generated by GSNC under laser light irradiation, the constructed Sa-M-GSNC killed the bacteria and significantly reduced the bacterial counts *in vitro* and *in vivo* (Wang et al., 2018).

**Figure 3:**
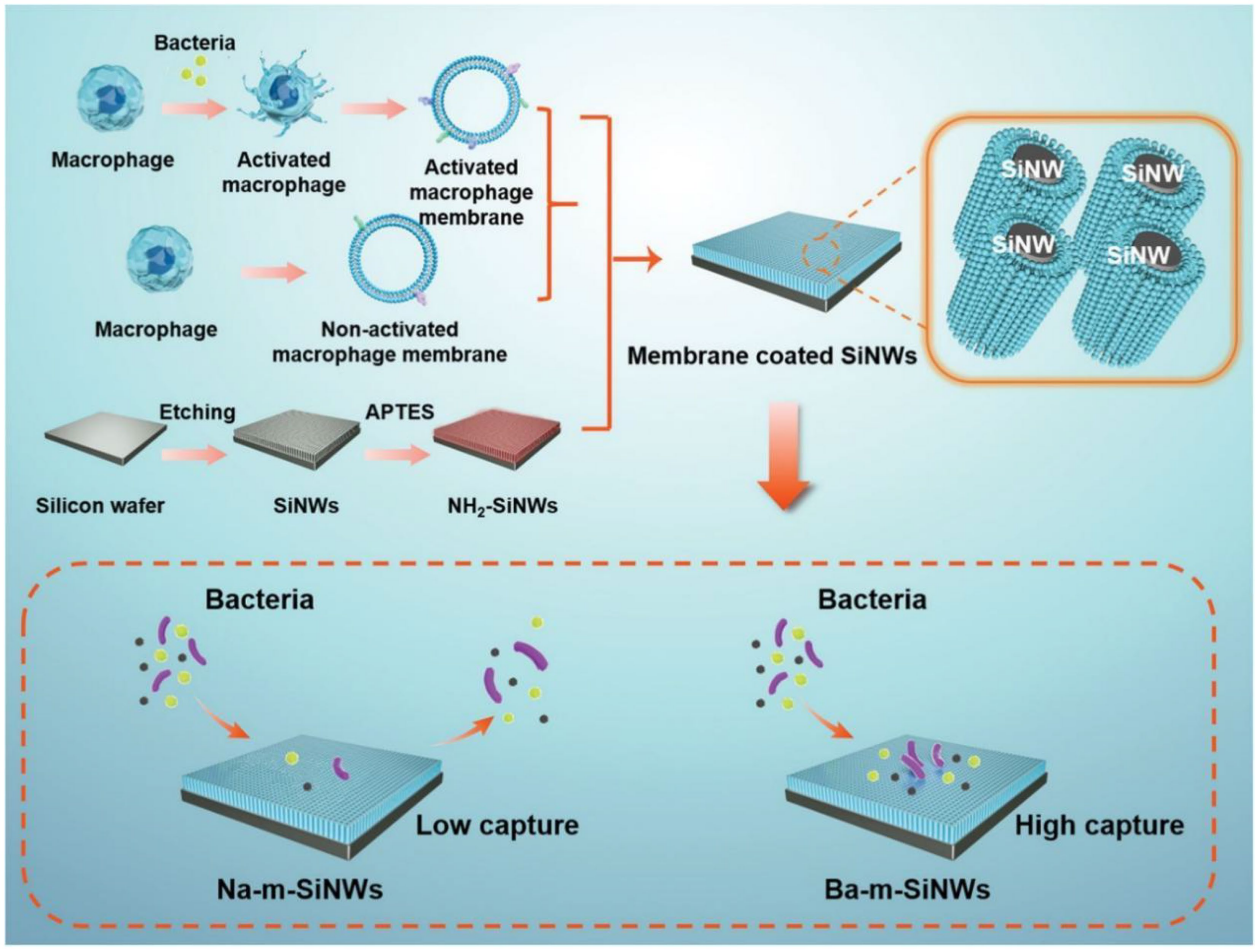
Schematic illustration of the preparation process of Na-m-SiNWs and Ba-m-SiNW. Ba-m-SiNWs exhibit more enhanced pathogen capture than Na-m-SiNWs. Reproduced with permission (Liu S. et al., 2021; Copyright© 2021, Wiley-VCH). Na-m-SiNWs, silicon nanowires coated by nonactivated macrophage membranes; Ba-m-SiNWs, silicon nanowires coated by bacterially activated macrophage membranes.

Clinically, intravascular catheters, mechanical ventilation equipment, hemodialysis machines, and other medical devices are all potential entry points for bacteria (Cecconi et al., 2018). The survival percentage of septic patients can be greatly improved by using antibiotics immediately after diagnosis (Font et al., 2020). Given the rising prevalence of multidrug-resistant bacteria, extracorporeal cleansing devices are increasingly attractive (Kang et al., 2014; Didar et al., 2015). Current extracorporeal blood purification systems rely on the adsorption or adherence of pathogen-associated molecular patterns and cytokines released by bacteria. Their therapeutic efficacy is inconsistent and unclear (Kang, 2020). Liu et al. reported a microfluidic device equipped with an interconnected nanowired silicon (Si) capture surface. To use macrophages’ inherent blood compatibility and ligand–receptor binding ability, they coated the membranes onto Si nanowire surfaces. Upon stimulation by *S. aureus* or *Escherichia coli*, the macrophages developed low negative zeta potentials, which allowed them to capture nonspecific bacteria. Additionally, the particular bacterial capture was aided by Toll-like receptors in bacterially activated membrane coatings on nanowired surfaces, which are missing in nonactivated membrane coatings. These two features, along with the maintenance of fluidity in activated membrane coatings, were responsible for the broad spectrum and high capture efficiency of all ESKAPE (*Enterococcus faecium, Staphylococcus aureus, Klebsiella pneumoniae, Acinetobacter baumannii, Pseudomonas aeruginosa*, and *Escherichia coli*) panel pathogens, which are regarded as the most threat to people health (Figure 3; Liu S. et al., 2021).

**Figure 4:**
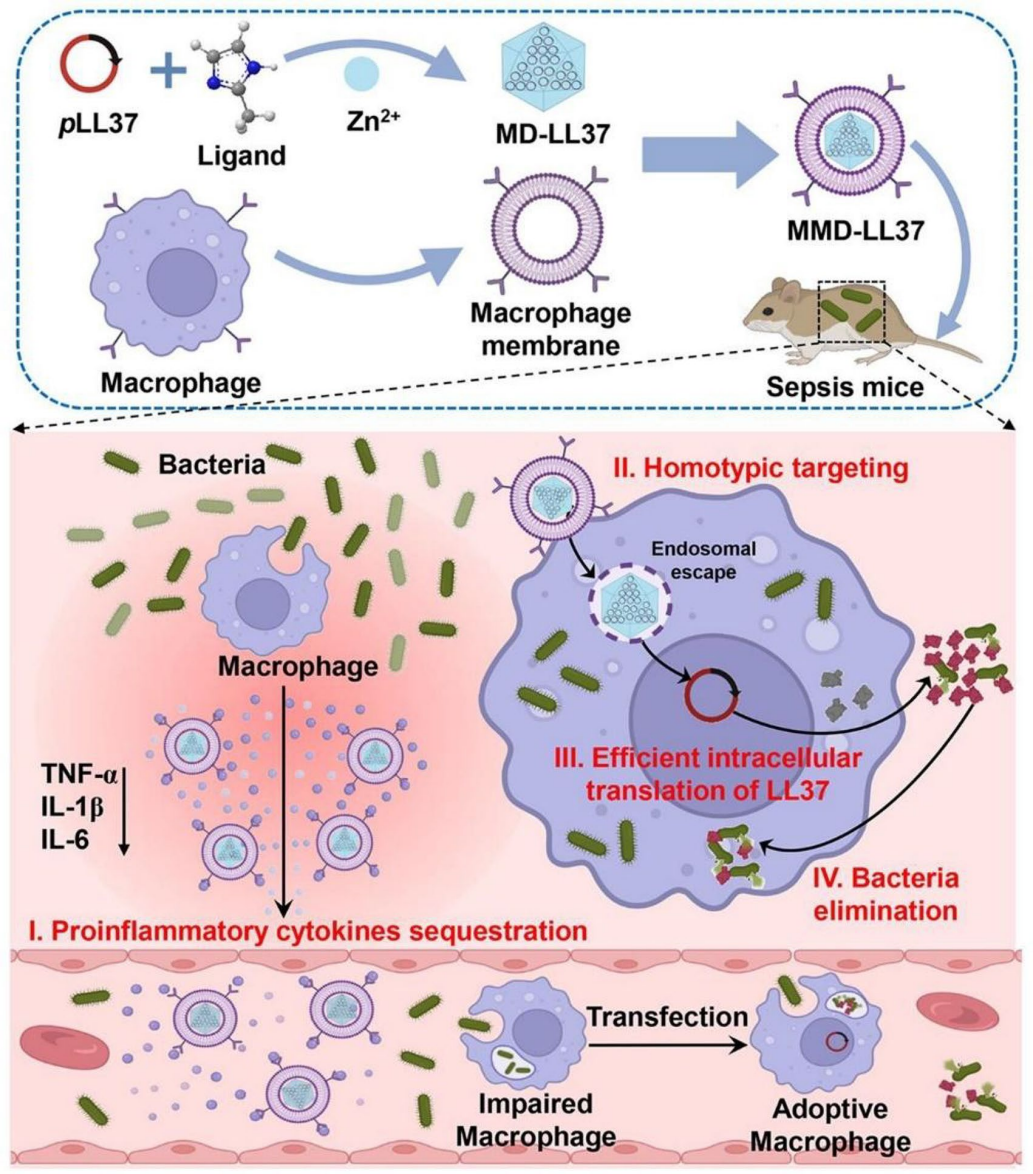
Schematic illustration of the application of macrophage membrane-coated metal–organic framework (MOF) for pLL37 delivery (MMD-LL37) and sepsis therapy. The constructed MMD-LL37 can sequester pro-inflammatory cytokines, realize the targeted delivery of plasmid, generate antibacterial LL37, and eradicate bacteria in circulation and those hidden inside cells. Reproduced with permission (Cao et al., 2022; Copyright© 2022, American Chemical Society).

Sepsis, caused by systemic bacterial infection, is a potentially fatal condition with widespread consequences (Landersdorfer and Nation, 2021). As the standard treatment for sepsis, antibiotic therapy encounteres challenges of drug-resistant bacteria (Dugar et al., 2020; Asner et al., 2021). Cao et al. reported a macrophage membrane–coated MOF system for delivering antimicrobial gene LL37 and fighting sepsis. LL37 can be delivered specifically to macrophages by coating macrophage membranes. This method facilitated the production of antimicrobial peptides in a continuous fashion. The constructed system significantly increased the survival rates of immunosuppressed septic mice infected with MRSA *via* effective gene therapy and sequester of inflammatory cytokines (Figure 4; Cao et al., 2022), demonstrating the potential of macrophage membrane coating in gene therapy. Soracha et al. reported a biomimetic nanoparticle composed of PLGA core and macrophage membrane surface. The developed nanoparticles were able to bind to endotoxins, absorb them, and prevent them from eliciting an immunological response. In addition, the nanoparticles mimicking macrophages captured proinflammatory cytokines and prevented them from amplifying the sepsis cascade. In a mouse *E. coli* bacteremia model, the obtained nanoparticles lowered the levels of proinflammatory cytokine, limited bacterial dissemination, and increased the survival rate (Thamphiwatana et al., 2017). Similarly, magnetic composite nanoparticles with osteoconductive Ca_3_(PO_4_)_2_ and antibacterial TiO_2_ were phagocytosed into macrophages for the preparation of membrane-coating nanoparticles and bone infection treatment. The obtained system displayed excellent properties in recognizing and absorbing bacteria, toxins, and inflammatory cytokines, thus exerting good antibacterial capacity *in vitro* and *in vivo* (Shi et al., 2021).

In addition to delivering genes, recognizing bacteria, and absorbing endotoxins, WBC-NPs can serve as carriers for antibiotic delivery. For example, bacteria “hide” in mammalian cells to avoid being killed by antibiotics and the host immune system (Weiss and Schaible, 2015). When *S. aureus* enter macrophages, the killing mechanisms of antibiotics might be inactivated, and this effect allow intracellular habitation and spread of infections (Peyrusson et al., 2020; Pidwill et al., 2020). Li et al. prepared a binary antimicrobial nanoparticle (ANP) consisting of triclosan and ciprofloxacin. Encapsulation within macrophage membranes improved the stability of the ANP (Li et al., 2020). The obtained macrophage membrane–encapsulated ANP, named Me-ANP, were superior to nonencapsulated ANP and clinically used ciprofloxacin in killing *S. aureus* in mice with peritoneal infection. Me-ANP were more effective than ciprofloxacin in eradicating organ infections caused by the spread of infected macrophages through the bloodstream in mice. The findings suggested a viable approach for therapeutic application in humans to tackle chronic infections.

Neutrophils play vital roles in fighting invading pathogens and can express more than 30 receptors capable of sensing inflammatory mediators (Németh et al., 2020). Through various mechanisms, including phagocytosis, degranulation, neutrophil extracellular traps, and reactive oxygen species–mediated damage, neutrophils can neutralize infectious threats (Petri and Sanz, 2018). Nanoparticles coated by neutrophil membranes are the essential components of WBC-NPs. For example, bioinspired microrobots that can actively move in biological fluids have attracted substantial interest for biomedical applications (Aziz et al., 2020). Zhang et al. attached neutrophil membrane-coated polymeric nanoparticles delivering ciprofloxacin to natural microalgae to obtain microrobots. The microrobots demonstrated quick speed in simulated lung fluid, homogeneous distribution, minimal clearance by alveolar macrophages, and excellent tissue retention duration after intratracheally administrated. Mice with acute *Pseudomonas aeruginosa* pneumonia receiving microrobots treatment exhibited a significant decrease in bacterial burden and remarkable increase in survival rate (Zhang et al., 2022). Neutrophil membrane–coated MOF loaded with glucose oxidase and chloroperoxidase were applied for the therapy of subcutaneous infection caused by *S. aureus* in mice. The developed nanoparticles, when injected intravenously, greatly decreased local bacterial load and wound size in comparison to uncoated MOF nanoparticles (Zhang et al., 2019).

### 3.3. Platelet membrane–coated nanoparticles

In the bloodstream, platelets—tiny and anucleate cell fragments—abound. Platelets are traditionally responsible for regulating blood clotting and the integrity of blood vessels. However, recent studies have shown that platelets serve as sentinel effector cells in the onset of bacterial infection (Jahn et al., 2022). Interactions between platelets and innate immunity play a significant role in responses to pathogens because platelets can sense and react to dangerous signals, and guide leukocytes to inflammation, damage, or infection sites (Yadav et al., 2019). Platelets can affect the recruitment and activation of immune effector cells by direct or indirect means (Palankar et al., 2018). In an intravascular infection, the first and most numerous cells to accumulate are platelets. Additionally, platelets can kill microorganisms by secreting antimicrobial peptides, such as defensins, cathelicidins, and kinocidins (Pircher et al., 2018; Kunde and Wairkar, 2021). Aggregated platelets can capture microbes and prevent their spread. Surface moieties specific to platelets mediate immunological evasion, subendothelial adhesion, and pathogen contacts (Imran et al., 2022a). Overall, platelets perform functions apart from inducing blood clotting and play a major role in regulating hosts’ immunological and inflammatory responses. In addition, platelet membrane-coating nanoparticles (PLT-NPs) can remarkably reduce macrophage uptake and complement activation and demonstrate platelet-like functions even after camouflaging, thereby solving the problem of plasma protein absorption on nanomaterial surfaces. Thus, esearch into PLT-NPs in infectious diseases has attracted widespread interest (as shown in Table 3; Kunde and Wairkar, 2021).

**Table 3:** Representative cell membrane coated nanoparticles for therapy of bacterial infection, in which cell membranes are derived from platelet, bacteria and other membrane.

Membrane type	Core materials	Functions	Pathogens	Infection model	References
Platelet membrane	PLGA nanoparticles	Neutralize *S. aureus* toxins; block damage to platelet and macrophage; preserve antibacterial capacity of host cells	MRSA	Systemic infection (i.p. injection of MRSA)	Kim et al. (2019)
Platelet membrane	Ag-MOF loading vancomycin	Target bacteria; kill MRSA through a comprehensive physical and chemical mechanism	MRSA	Pneumonia	Huang et al. (2021)
*S. aureus-*derived EV	PLGA nanoparticles loading vancomycin or rifampicin	Targeting delivery of antibiotics; eliminate intracellular *S. aureus*	*S. aureus*	Bacteremia	Gao et al. (2019)
*E. coli-derived* OMVs	MSNs containing rifampicin	Improve targeting efficiency and enhance uptake of rifampicin; enhance antimicrobial activity	*E. coli*	Peritonitis	Wu S. et al. (2021)
Hybrid RBC membrane and PMB-modified liposome	PMB	Neutralize both endotoxins and exotoxins; anchor *E. coli*; realize target delivery	*E. coli*	Subcutaneous infection; intravenously injection of toxin;	Jiang et al. (2021)
Gastric epithelial cell membrane	PLGA nanoparticle loading clarithromycin	Specifically adhere to bacteria; realize targeted delivery of antibiotics	*H. pylori*	Infection caused by oral gavage of bacteria	Angsantikul et al. (2018)
Dendritic cell membrane	CuFeSe_2_ nanoparticle	Pathogen-binding and immune-evading ability; kill bacteria under NIR irradiation; targeted delivery to infected site	*S. aureus*	Subcutaneous abscess mouse model	Hou et al. (2021)
Genetically engineered cell membrane	Sonosensitizer	Targeting pathogenic bacteria, neutralize exotoxin, diagnosis of pathogenic bacterial exotoxin infection; generate ROS to kill bacteria	MRSA	Systemic infection (tail vein injection of MRSA)	Pang et al. (2019a)

PLGA, poly(lactic-co-glycolic acid); *S. aureus*, *Staphylococcus aureus*; i.p., intraperitoneal; MRSA, methicillin-resistant *Staphylococcus aureus*; MOF, metal–organic framework; EV, extracellular vesicles; *E. coli*, *Escherichia coli*; OMVs, outer membrane vehicles; MSNs, mesoporous silica nanoparticles; PMB, polymyxin B; *H. pylori*, *Helicobacter pylori*; NIR, near-infrared; ROS, reactive oxygen species.

Among the most common opportunistic gram-positive bacterial pathogens, *S. aureus* (especially MRSA), lead to a wide range of illnesses, such as sepsis, bacteremia, and endocarditis (Cheung et al., 2021). The production of toxins is the driving force behind *S. aureus* pathogenesis. Platelets express disintegrin and metalloproteinase domain-containing protein 10, a receptor of α-toxin, on surface membranes (Surewaard et al., 2018). Thus, platelets are the targets of *S. aureus* as well. Kim et al. constructed biodegradable nanoparticles consisting of PLGA cores and biomimetic platelet membranes for blocking the cytotoxicity of *S. aureus* and protecting the body from lethal systemic infection. By inhibiting platelet damage caused by *S. aureus* toxin secretion, the nanoparticles stimulated platelet activation and acted as bactericides. In a similar fashion, the nanoparticles counteracted *S. aureus-*secreted toxin-induced macrophage damage. Thus, MRSA-induced neutrophil extracellular trap release was reduced, and the bactericidal, oxidative burst, and nitric oxide generation of macrophages were bolstered. Therapy with the developed nanoparticles decreased bacterial counts in the blood and reduced mortality in MRSA-induced systemic infection in mice. The findings offered demonstrative evidence of the therapeutic efficacy of PLT-NPs in neutralizing toxin, providing cytoprotection, and enhancing resistance to infection (Kim et al., 2019).

In another study, 2-methylimidazole and silver nitrate were used in preparing a nanosilver MOF for Van loading. Ag-MOF-Vanc were encapsulated by nano-sized platelet vesicles to obtain PLT@Ag-MOF-Vanc. PLT@Ag-MOF-Vanc outperformed free Van against a panel of typical clinical pathogens. The mechanisms of PLT@Ag-MOF-Vanc killing bacteria is multifaceted and involves disturbance of bacterial metabolism, catalysis of reactive oxygen species generation, disruption of cell membrane integrity, and inhibition of biofilm formation. The platelet membrane allowed PLT@Ag-MOF-Vanc to attach to MRSA and infectious sites. In a mouse model of MRSA pneumonia, PLT@Ag-MOF-Vanc showed a strong anti-infective effect that was significantly more effective than free Van and did not cause any evident harm (Huang et al., 2021).

Phage treatment potentially mitigates antibiotic resistance (Kortright et al., 2019). The short half-lives of bacteriophages in the bloodstream are the largest obstacles to their widespread use. Jin et al. reported a blood circulation-prolonging peptide (BCP1) with an RGD motif binding to integrins; the peptide improved circulation by interacting with platelets (Jin et al., 2021). Researchers have developed biomimetic phage–platelet hybrid nanoparticles (PPHNs), aiming to extended the use of BCP1 in bacterial infection. The obtained PPHNs with a particle size of ~350 nm showed sustained antimicrobial capacity and increased blood retention time to combat *E. coli* infection *in vivo*, demonstrating more effective antibacterial properties prophylactically and therapeutically when compared with BCP1 (Jin et al., 2021). This investigation on hybrid membrane further expanded the use of platelet membrane and provided a novel strategy for applying phage-based nanoparticles to bacterial infection treatment.

### 3.4. Bacterial membrane–derived nanoparticles

Bacterial MVs are particles with diameters of 20–400 nm and are secreted by bacteria (Sartorio et al., 2021). Gram-negative and gram-positive bacteria can secret MVs into the extracellular environment. MVs from gram-positive bacteria are called extracellular vesicles (EVs), and those from gram-negative bacteria are outer membrane vehicles (OMVs) (Toyofuku et al., 2019). MVs carry a diverse range of immunogenic antigens and pathogen-associated molecular patterns essential for regulating host immune responses, and allow the immune system to respond either broadly or specifically (Behrens et al., 2021). Integrating gold nanoparticles with *E. coli*-secreted OMVs provides nanovaccine capable of eliciting significantly increased immune responses in comparison to intact *E. coli* OMVs (Gao et al., 2015), indicating that the immunomodulatory function of antigens on the OMV surface are unaffected by the membrane-coating technology (Brown et al., 2015). Immunostimulatory property and proteoliposome nanostructure make MVs attractive candidates for vaccines or delivery systems against bacterial infections (Sartorio et al., 2021). Examples of bacterial membrane–derived nanoparticles’ applications are shown in Table 3.

**Figure 5:**
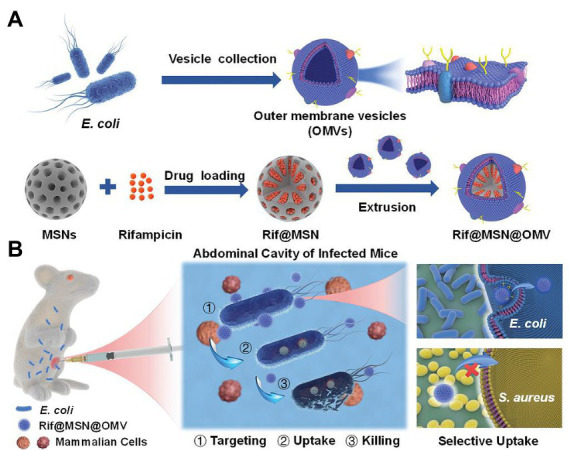
Schematic illustration of **(A)** the preparation process and **(B)** the application of OMV-coated biomimetic nanodelivery system Rif@MSN@OMV in bacterial infection. OMV modification achieved homotypic targeting capacity and enhanced uptake, thus exerting strong antibacterial activity. Reproduced with permission (Wu S. et al., 2021; Copyright© 2021, Wiley-VCH) OMV, outer membrane vehicles; Rif, rifampicin; MSN, mesoporous silica nanoparticles; *E. coli*, *Escherichia coli*.

OMVs-based nanodrug delivery systems have been reported. Wu et al. coated mesoporous silica nanoparticles (MSNs) encapsulating rifampicin (Rif) with OMVs isolated from *E. coli.* Owing to their homotypic targeting activity, OMVs significantly enhanced MSN absorption in *E. coli* but not in gram-positive *S. aureus*. Compared with free Rif with only modest bactericidal activity, Rif@MSN@OMV with excellent biocompatibility totally eradicated bacteria at a comparable Rif concentration. Moreover, Rif@MSN@OMV increased the survival rates of mice with peritonitis induced by *E. coli* and significantly reduced the bacterial load in the intraperitoneal fluid and related organs (shown in Figure 5; Wu S. et al., 2021). The premature release of encapsulated antibiotics was inhibited, and the internalization process by the same bacteria was significantly enhanced by membrane coating. In theory, this method increases the susceptibility of gram-negative bacteria to medications that have been historically ineffective.

Most complications of *S. aureus* bacteremia result from the pathogen’s ability to survive within host phagocytes, particularly macrophages. Eliminating the intracellular *S. aureus* is fundamental for clinical success (Foster, 2005). In addition to research focusing on macrophage membrane-coated nanoparticles (Li et al., 2020), the use of EVs in bacterial infection treatment has been explored. Gao et al. coated antibiotic-loaded nanoparticles (NP-antibiotic) with EVs secreted by *S. aureus* to prepare an active targeted delivery nanosystem. NP-antibiotic@EV served as a “Trojan horse,” bringing antibiotics into infected macrophages and subsequently killing intracellular *S. aureus.* The results demonstrated that NP-antibiotic@EV is as effective as encapsulated antibiotics or more effective despite the prolonged release behavior. After intravenous administration to mice bearing *S. aureus,* NP@EV achieved active and targeting distribution in organs suffering from metastatic infections. Moreover, NP-antibiotic@EV enabled the encapsulated antibiotic to exert remarkably enhanced anti-infection capacity (Gao et al., 2019).

Dental caries is a chronic, progressive, and devastating disease caused primarily by bacterial infection cariogenic biofilms. Cariogenic bioflms are difficult to treat because bacterial pathogens like *Streptococcus mutans* reside in a self-producing matrix of extracellular polymeric substances (EPS). The penetration and retention of antibiotics into biofilms is severely deficient. To solve this problem, membrane derived from *Lactobacillus acidophilus* was utilized to coat PLGA nanoparticles encapsulating triclosan (TCS), in which *L. acidophilus* was capable of inhibiting the colonization and bioflm formation of *S. mutans via* the coaggregation with *S. mutans.* The resulting biomimetic nanoparticles, LA/TCS@PLGA-NPs, have the following advantages: (1) By viture of the native properties of *L. acidophilus,* LA/TCS@PLGA-NPs can adhere to *S. mutans* and hinder the formation of *S. mutans*’ biofilm; (2) LA/TCS@PLGA-NPs can incorporate into the biofilm, and serve as a depot for sustained antibiotic release to prevent the spread of *S. mutans* biofilm (Weng et al., 2022). This study offers novel insights into CM-NPs for combating bacterial biofilms and associated illnesses.

### 3.5. Other cell membrane–coated nanoparticles

Membranes from RBC, WBC, PLT, and bacteria have been combined with nanomaterials. The procedure prevents clearance by the immune system and extends retention time, but whether the prepared biomimetic nanomaterials can specifically target specific cells remains a pressing issue (Pang et al., 2019a). Hybrid cell membranes have recently attracted attention because they potentially improve the functionality of biomimetic nanoparticles (Jiang et al., 2021). For example, a polymyxin B (PMB)-modified RBC-biomimetic hybrid liposome (P-RL) was developed for antivirulence therapy of *E. coli* infection. The interaction between PMB and *E. coli* membrane contributed to the attachment and anchoring of P-RL to *E. coli.* The fusion of the RBCM and the modified PMB allowed P-RL to effectively neutralize endotoxins and exotoxins (Table 3; Jiang et al., 2021). In another study, a hybrid of RBC membrane and PLT membrane was applied to functionalize nanorobots for the simultaneous elimination of bacteria and toxins (Esteban-Fernández de Ávila et al., 2018).

**Figure 6:**
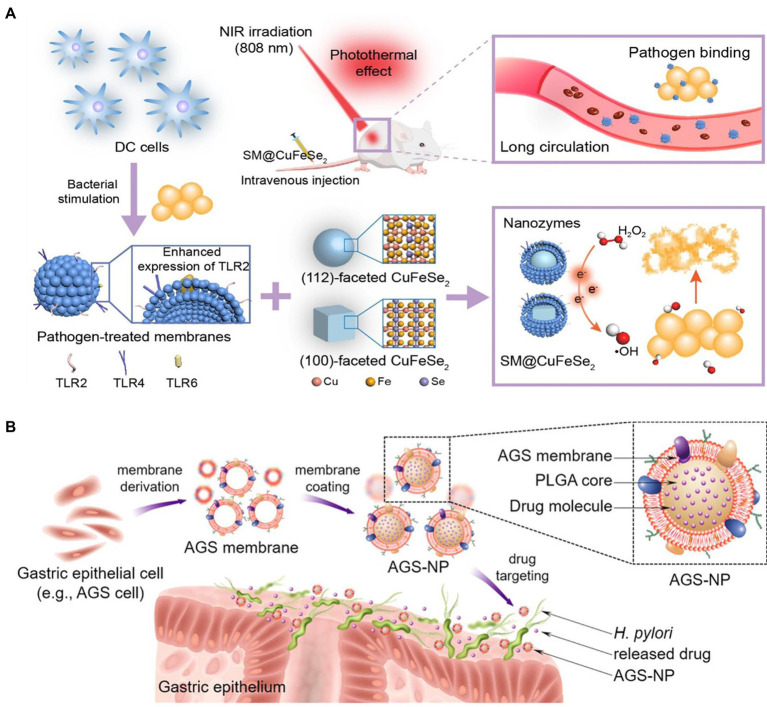
**(A)** Construction of dendritic cell (DC) membrane coated CuFeSe2 nanocrystals and their therapeutic application with NIR in bacterial infection (TLR, SM@CuFeSe_2_ and NIR referred to toll-like receptor, pathogen-pretreated membrane-coated CuFeSe_2_ and near-infrared, respectively). Reproduced with permission (Hou et al., 2021; Copyright© 2021, American Chemical Society). **(B)** Schematic illustration of the developed of gastric epithelial cell membrane-coated PLGA nanoparticles’ application in targeted antibiotic delivery for treating *Helicobactor pylori* infection. Reproduced with permission (Angsantikul et al., 2018; Copyright© 2018, Wiley-VCH).

As well-known antigen-processing cells, dendritic cells (DCs) play a crucial role in triggering innate and acquired immunological response after bacterial recognition *via* Toll-like receptors (Adib-Conquy et al., 2014; Bieber and Autenrieth, 2020). Hou et al. used *S. aureus*-pretreated DC membrane (SM) to coat CuFeSe_2_ nanocrystals that are highly efficient in converting light into heat. The resulting complex, SM@NC, efficiently bind to *S. aureus* and exhibited stealthy immune evasion and targeted delivery to the infectious site (Figure 6A). The membrane coating remarkably enhanced the antibacterial capacity of the CuFeSe_2_ nanocrystals. In combination with near infrared irradiation, the intravenous administration of SM@NC significantly reduced the bacterial colonies in infected tibia (Table 3). These findings provided proof of concept for the therapeutic application of pathogen receptor membrane-coated nanoparticles to the treatment of infectious diseases (Hou et al., 2021).

*Helicobacter pylori* infection is a primary cause of pepticulcer illness, inflammatory gastritis, and gastric cancer, creating a global healthcare burden (Fischbach and Malfertheiner, 2018). A combinational therapy including a proton pump inhibitor and antibiotics, such as clarithromycin and amoxicillin, has been widely recommended for *H. pylori* infection treatment (de Brito et al., 2019). However, increasing drug resistance often results in therapy failure. Indeed, novel and effective strategy is urgently needed. Inspired by the cell membrane-coated nanoparticles, Angsantikul et al. developed gastric epithelial cell membrane-coated nanoparticle for delivering antibiotics against *H. pylori* based on the specific binding between *H. pylori* and gastric epithelial cell (Figure 6B). With the feature of sharing the same antigens on surfaces with the original AGS cells, the obtained nanosystem can naturally adhere to *H. pylori* bacteria. AGS-NPs demonstrated specific accumulation on bacterial surface. The clarithromycin (CLR)-encapsulated AGS/NP exhibited markedly improved antibacterial efficacy compared with free CLR and nontargeted nanoparticles. These findings highlighted the potential of employing the membrane of native host cells significantly related to the specific pathogens to functionalize nanocarriers (Angsantikul et al., 2018).

Genetically engineered cell membranes have also been applied to coat nanomaterials and used in bacterial infection treatment. Genetic membrane engineering was utilized to endow HEK293T cells with the capacity of expressing specific antibody MEDI4893, a monoclonal antibody (MAb) that specifically neutralizes alpha-toxin of MRSA (Kong et al., 2016). Subsequently, MAb-piloting nanovesicles (ANVs) were applied to encapsulate sonosensitizer (Pang et al., 2019a). The extremely active antibody–toxin interaction made the ANVs more effective in capturing toxins than traditional passive neutralizing toxin capacity endowed by natural RBCM. Sonosensitizers, when activated by ultrasound, produce reactive oxygen species that efficiently kill bacteria and accelerate virulence clearance (Pang et al., 2019b). By eradicating bacteria and neutralizing pathogenic toxins simultaneously, the reported nanomaterial potentially prevents and controls infection caused by multidrug-resistant bacteria. Moreover, when guided by an antibody, a nanocapturer may precisely pinpoint MRSA infection and differentiate it from benign inflammation. As the first of its kind, this novel approach combines antibacterial sonodynamic therapy with antivirulence immunotherapy, opening the door to antibiotic-free nanotheranostics that can effectively combat multidrug-resistant bacterial illnesses.

Extracellular vesicles (EVs) released by cells are nano-sized MVs that have proven to be highly effective in the development of biomimetic nanoplatforms (Herrmann et al., 2021). In comparison to EVs made from mammalian cells, plant-derived edible EVs with low immunogenicity are environmentally friendly, sustainable, and amenable to large-scale production. Combining ginger-derived EVs and Pd-Pt nanosheets, Qiao et al. yielded a biomimetic nanoplatform (EV-Pd-Pt) for synergistic bacteria and biofilm elimination. Ginger-derived EVs helped EV-Pd-Pt stay in the blood longer without being cleaned by the immune system and accumulate at infection sites. EV-Pd-Pt got access to the interior of bacteria *via* an EV lipid-dependent means. Remarkably, electrodynamic and photothermal therapies mediated by EV-Pd-Pt nanoparticles exhibited synergistic benefits and great efficiency in elimination of *S. aureus* biofilms (Qiao et al., 2022).

## 4. Discussion: Perspectives and challenges

Most nanomaterials require surface modification before application in biomedical settings, but conventional approaches have been shown to be unsatisfactory (Nemani et al., 2018). Based on the many roles of the cell membrane, cell membrane coating is a sought-after alteration to bestow nanomaterials with excellent biological interface capabilities. Membrane components, such as membrane proteins, polysaccharides, and lipids, are moved to the surfaces of nanoparticles, affording CM-NPs the intrinsic activities and properties of the derived cells (Fang et al., 2018). These CM-NPs can be used for various purposes, including neutralizing toxins, evading clearance by the immune system, targeting specific bacteria, delivering antibiotics, and regulating immune responses (Ma et al., 2022). The recent advances in applications of CM-NPs in bacterial infection are listed in Tables 1–3. Owing to the strategy’s adaptability, various membranes and nanoparticle cores can be combined to serve a variety of therapeutic goals. Through genetic engineering, cell membranes can acquire abilities not found in their source cells (Pang et al., 2019a). These methods provide hope for the integration of numerous antimicrobial components into multifunctional CM-NPs by the simultaneous expression of multiple proteins on the cell membrane surface and for effective therapy against bacterial infection.

Furthermore, antibiotic-free therapeutic strategy attracts considerable interest in the context of post-antibiotic era. Compounds of natural origin with antibacterial capacity extracted from plant, animal, bacteria and so on, are of tremendous scientific interest as potential therapeutic tools (Álvarez-Martínez et al., 2020). For example, tannins, found in diverse plants such as mimosa, chestnut, quebracho, display good antimicrobial and anti-biofilm effects against various bacteria, and are potential alternatives to conventional antibiotics. Mesoporous silica, hyaluronic acid (HA), gelatin, and chitosan nanocarrier were utilized for delivering tannins (Farha et al., 2020). The nanoparticles can be further modified by cell membrane to enhance the antibacterial capacity of tannins. CM-NPs may be also used in conjunction with photodynamic, sonodynamic, and photothermal therapies to overcome the challenges posed by the photosensitizers’ and sonosensitizers’ potential instability in circulation and lack of targeted distribution (Lin et al., 2019; Pang et al., 2019a; Chen et al., 2021). In addition, by optimizing the fabrication of antibiotic-loaded CM-NPs, we may combine the antibiotics’ unique PK/PD indices to boost their anti-infective efficiency by regulating drug release behavior. Currently, the application of CM-NPs in bacterial infection mainly focuses on MRSA and *E. coli.* Massive research is required to confirm their effectiveness in combating multidrug-resistant gram-negative bacteria, such as *Klebsiella pneumoniae, Acinetobacter baumannii*, and *P. aeruginosa*.

Despite the numerous benefits of CM-NP for bacterial infection, their use still faces numerous obstacles. First, from the point of view of CM-NP preparation, the uniformity of CM-NPs can be affected by the donor cell type. Medicinal efficacy may vary from batch to batch because of the unique characteristics of cell membranes at different stages of growth during the cell cycle. The whole process of preparing CM-NPs needs to be conducted in a sterile setting. If a desired product is contaminated by bacteria, infection symptoms and diseases will worsen after administration (Ma et al., 2022). Overcoming these obstacles should be possible with the imminent implementation of efficient workflow and appropriate quality control assays (Imran et al., 2022a). Second, the insufficient supply of numerous cells and the inefficiency of cell membrane extraction procedures have posed obstacles to large-scale production (Le et al., 2021). Third, from the application standpoint, unwanted side effects may be induced by the membrane modifications necessary to create multifunctional CM-NPs. The overuse of CM-NPs can cause or exacerbate inflammation *via* interaction with the immune system and results in the release of pathological mediators (Fang et al., 2018). Nanoparticles with maximal simplicity and functionality should be further explored.

## 5. Conclusion

In conclusion, we briefly summarized the recent advances in applications of cell membrane–coated nanoparticles in bacterial infection. Therapeutic biomimetic nanosystems consist of cell membranes, and nanoparticles constitute an exciting field for bacterial infection treatment. We expect that advances in this technology will improve methods for treating bacterial illnesses and reducing threats posed by bacteria resistant to antibiotics despite the abovementioned obstacles.

## Author contributions

YS contributed to the conceptualization and wrote the original draft. XZ reviewed and edited the manuscript. JH, SM, KL, and JC performed literature research and organized the database. XX supplied guidance and revised the draft. XL and XW provided the funding support and revised and edited the draft. All authors contributed to the article and approved the submitted version.

## Funding

The research was supported by National Natural Science Foundation of China (82003660 and 81971981), the Nature Science Foundation of Zhejiang province (LQ20H300003 and LYY21H300004), Zhejiang Pharmaceutical Association Hospital Pharmacy Special Scientific Research Funding Project (2019ZYY02) and Zhejiang Medical and Health Science and Technology Plan Project (2019RC170).

## Conflict of interest

The authors declare that the research was conducted in the absence of any commercial or financial relationships that could be construed as a potential conflict of interest.

## Publisher’s note

All claims expressed in this article are solely those of the authors and do not necessarily represent those of their affiliated organizations, or those of the publisher, the editors and the reviewers. Any product that may be evaluated in this article, or claim that may be made by its manufacturer, is not guaranteed or endorsed by the publisher.
